# Atlanta Society of Medicine

**Published:** 1894-04

**Authors:** J. W. Duncan

**Affiliations:** President, in the Chair


### Society Reports.

### ATLANTA SOCIETY OF MEDICINE.

MEETING FEBRUARY 19, 1894.

*President J. W. Duncan in the Chair.*

### REPORT OF CASES.

DR. KIME: *Case 1.* Mrs. H., primipara, wife of a physician, age twenty-one years. Taken with "Grippe" on Saturday, confined on Monday, developed pneumonia Monday night, lower portion of left lung involved.

During labor, pains being inefficient, as much as twenty (20) grains of quinine was given, otherwise labor normal, some chloroform used.

Twenty to twenty-five grains of quinine were administered for three consecutive days, being combined with a small amount of calomel triturate and dover's powder. To allay cough, a mixture containing syrup of squills, tincture aconite, paregoric and syrup of wild cherry was administered for two days. After second day was kept upon a mixture containing nux vomica, tincture digitalis, tincture of cinchona compound and essence of pepsin and the cough mixture changed to one containing carbonate of ammonia.

A binder was placed on patient with a folded cloth fastened to it behind and in front, passing between the thighs to hold an antiseptic occlusion pad on vulva. Patient made an uneventful recovery.

*Remarks:* This is the fourth case of pneumonia complicating parturition occurring in my practice. All recovered, which I am inclined to believe was a result of precautions taken.

I think the most important points in the treatment of these cases is to prevent excessive motion of bowels and air entering vagina, when the patient is compelled to cough. This is accomplished by quieting cough as much as possible by medication, then pinning broad abdominal binder snugly around patient with an antiseptic vulval occlusion pad held in position by a folded cloth pinned to binder in front and behind.

The dressing not to be removed as long as cough continues only to allow bowels to move, kidneys to act and to cleanse parts. At first the parts should be cleansed three or four times in twenty-four hours with a warm solution of mercuric chloride 1 in 2 to 3,000, not allowing any discharges to remain long enough to decompose; pad should be renewed as soon as soiled, the one removed being burnt.

Absorbent cotton with free use of boric acid, makes the best pad. Never use vaginal douches until discharges begin to decompose or become offensive or infection occurs, then use them, and if necessary, the intra-uterine douche as well.

Ordinarily one of the cardinal points for success in the treatment of pneumonia is to sustain vital powers of patient by improving digestion with systematic feeding. In these cases it is far more important, for we have in addition to the pneumonic inflammation the drain on vital powers in lactation, lochial discharge and absorption from involution of generative organs.

To stimulate the flagging energies, breathing center and tide over the critical period, we have nothing equal to strychniam-hypodermatically, or per orem if stomach can assimilate it.

DISCUSSION.

DR. MCRAE indorses the use of strychnia, one of the best we have in pneumonia, better than digitalis, does not contract arterioles like digitalis.

DR. GLASS condemns digitalis in pneumonia, but indorses the use of strychnia.

DR. BAIRD uses digitalis in pneumonia with benefit, would not like to be without it, believes it strengthens action of the heart. Also indorses use of strychnia. Often uses as a stimulant camphor dissolved in ether, gives it hypodermically—it is transient in effect, not a tonic, but stimulant—strychnia and digitalis more prolonged in effect.

DR. E. VAN GOIDTSNOVEN indorses the use of strychnia and digitalis. Uses digitaline in $\frac{1}{100}$ grain doses, four hours.

DR. RICHARDSON indorses digitalis in pneumonia, and is pleased with its effect.

DR. BAIRD reported case of a boy twelve years old; temperature $103\frac{1}{2}°$ F.; pulse 120 with pain, tenderness, and swelling in region of cæcum size of an orange, with symptoms indicating inflammation. Gave calomel and salines for three consecutive days, after which he passed two large fecal masses, with immediate improvement.

DR. AMSTER reported a case of carbuncle. Same patient had one two years previously, treated by crucial incision with others forming at each end of incisions. Patient refused to have this one so treated. Wished suggestions as to best methods of treatment without operation.

DR. GLASS suggested use of carbolic acid with glycerine used as an injection, which he had used with benefit. Used it after reading an article by Dr. Crowe in *Atlanta Medical and Surgical Journal.*

DR. CROWE had used it in eight cases with success. Had one case of an old lady which he deemed removal by incision and curetting best to prevent the extensive absorption. Thought the injection the best method in most all cases. Also keeps center well open for free drainage. The injections relieve pain. Objects to free use of whisky when patient can take plenty of nourishment. Uses a per cent. solution of carbolic acid.

THE PRESIDENT indorses carbolic injections—uses as strong as fifty per cent. solutions. Uses plain ginger poultice with great relief of pain. Changed every three to four hours.

DR. FLOYD W. MCRAE practices enucleation but not so extensive as Dr. Crowe advised. Has been trying ichthyol, and would advise its use in such cases.

Reported a case of syphilitic infection illustrating how innocent ones so often suffer. A child had contracted syphilis from nurse. A very small chancroid on lip, which was not characteristic enough to warrant a diagnosis; the mother became infected on nipple from the child nursing. Had seen chancroid on lips of a virgin.

DR. CROWE: This is an extremely important subject. May come home to any of us. Should be agitated. A large per cent.

of the colored race is syphilitic. In clinics, finds it very common—one in three cases. Should be brought before the public.

DR. VAN GOIDTSNOVEN reported a case where a physican contracted it from an obstetric case and infected his own wife. Thinks 75 per cent. of colored people have syphilis, scrofula or consumption.

DR. MCRAE stated Dr. Joseph Price had said, "any man with a chancre on his penis that would not control himself, should be be in prison." Would advise waiting for secondary symptoms before constitutional treatment, when in doubt.

DR. GLASS advised waiting until rash appears before giving treatment. That eruption will appear in time and hair fall out, even when treatment is commenced early.

DR. AMSTER does not believe secondary symptoms will appear when case is treated early.

DR. BOSWORTH, by invitation, reported a case that called to be treated for conjunctivitis which, on closer investigation, proved to be syphilitic. So treated, recovered.

PRESIDENT had seen case Dr. McRae referred to. Thought the public should be better informed on this subject. Does not treat syphilis until secondary symptoms appear, then treats heroically. Believes it to be very common in colored race.

MARCH 6, 1894.

*President J. W. Duncan, M. D., in the Chair.*

REPORT OF CASES.

DR. J. MCF. GASTON, SR., reported a case of smallpox on Ella street; confluent form, being unmodified, as patient had not been vaccinated. Called again later to see case and found it running the regular course of smallpox. The remainder of the family were vaccinated when case was diagnosed, and had varioloid. Had seen a case of varioloid with Dr. Hurt in another part of the city. Believes the eruption of varioloid to be very variable as to character and course.

DR. CHILDS reported a case of carbuncle treated by carbolic acid injections with good results. Thinks it the best plan of treatment. That carbuncle is due to a germ, and is destroyed by the carbolic acid. Does not desire suppuration.

DR. GASTON, SR., cauterizes carbuncle with nitric acid and applies adhesive plasters over it; as they get loose, reinforces them with more plaster. Thinks it favors the formation of pus, then they get well rapidly, and thinks it the best method of treatment.

DR. EARNEST presented a specimen of extra-uterine pregnancy. In January, 1894, patient called at his office. She had irregular flow in December and missed flow in January. On examination found enlargement near left cornea of uterus. Told her I thought she was pregnant. Saw no more of her until a few nights since. She had a severe attack of pain with some hemorrhage. Examination revealed a case of tubal pregnancy, with some peritonitis. Her husband being away, telegraphed for him. Operated, median incision, with but little hemorrhage, using a drainage tube for three hours. Then removed it before leaving patient. The uterus about three inches in depth, no decidual membrane. About seven years since last child was born.

